# Using Type I Collagen Gel to Prevent Postoperative Intrauterine Adhesion: A Multicenter Retrospective Study

**DOI:** 10.3390/jcm12113764

**Published:** 2023-05-30

**Authors:** Kwang Beom Lee, Seung Joo Chon, Sunghoon Kim, Dae Yeon Kim, Chan Woo Park, So Jin Shin, Seok Mo Kim, Ki Hwan Lee, Yong Il Ji

**Affiliations:** 1 Department of Obstetrics and Gynecology, Gachon University Gil Medical Center, Gachon University College of Medicine, Incheon 21565, Republic of Korea; 2 Department of Obstetrics and Gynecology, Women’s Cancer Clinic, Institute of Women’s Life Science, Yonsei University College of Medicine, Seoul 03722, Republic of Korea; 3 Department of Obstetrics and Gynecology, University of Ulsan College of Medicine, Asan Medical Center, Seoul 05505, Republic of Korea; 4 Department of Obstetrics and Gynecology, Fertility Center of CHA Gangnam Medical Center, CHA University School of Medicine, Seoul 13496, Republic of Korea; 5 Department of Obstetrics and Gynecology, Keimyung University School of Medicine, Daegu 42601, Republic of Korea; 6 Department of Obstetrics and Gynecology, Chonnam National University Medical School, Gwangju 61469, Republic of Korea; 7 Department of Obstetrics and Gynecology, Chungnam National University Hospital, Chungnam National University College of Medicine, Daejeon 34134, Republic of Korea; 8 Department of Obstetrics and Gynecology, Inje University, Haeundae Paik Hospital, Busan 48108, Republic of Korea

**Keywords:** adhesion, collagen, hysteroscopy, intrauterine

## Abstract

We evaluated the clinical outcomes of using type 1 collagen gel after therapeutic resectoscopy; overall, 150 women aged > 20 who planned to undergo therapeutic resectoscopy were enrolled. The patients were randomly assigned to either of the anti-adhesive treatment groups: the type 1 collagen gel (Collabarrier^®^) (study group; N = 75) or the sodium hyaluronate and sodium carboxymethylcellulose gel group (control group; N = 75) after resectoscopy. One month after applying anti-adhesive materials, postoperative intrauterine adhesions were evaluated using second-look hysteroscopy; the incidence rate of postoperative intrauterine adhesions examined through second-look hysteroscopy showed no significant differences between the groups. There were no statistical differences between the frequency and mean scores of the type and intensity of adhesions in both groups. Finally, no significant differences in adverse events, serious adverse events, adverse device effects, and serious adverse device effects were noted between the two groups; type 1 collagen gel can be effectively and safely used in intrauterine surgery to minimize postoperative adhesions, thereby eventually decreasing the prevalence of infertility, secondary amenorrhea, and recurrent pregnancy loss in reproductive women.

## 1. Introduction

Adhesion is the tendency of different cellular surfaces to cling to one another after various insults or physiological changes, such as surgery, inflammation, injury, or infection. Among these causes, surgical wounds seem to be the most concerning cause of adhesion [1]. During surgical wound healing, platelets, fibroblasts, and other substances create fibrotic bands, which cause organ adhesion [2]. Postoperative adhesion in organs and tissues is natural; however, it can lead to chronic pain or infertility in specific sites such as the pelvic cavity. Postoperative pelvic adhesion after abdominal and pelvic surgery occurs in approximately 70–90% of cases. It has been reported to occur especially after myomectomy, cystectomy, and surgery for endometriosis or intra-abdominal infection. It is clinically relevant, as it can progress to small bowel obstruction and infertility [1]. Moreover, during gynecological surgery, adhesions occur in the intra-abdominal, pelvic and uterine cavities [2]. After suctioning and evacuating the inside of the uterus, endometrial synechiae can occur in approximately 20–50% of cases, leading to infertility, amenorrhea, or recurrent pregnancy loss [3].

As postoperative adhesions induce various complications, several trials have been conducted to minimize these adhesions through minimal tissue manipulation during surgery. The development of surgical techniques has decreased the occurrence of postoperative adhesion by minimizing trauma, exposure to foreign materials, and dryness of tissues. However, there are fundamental limitations to preventing postoperative adhesion using surgical methods. This has prompted the development of other methods, including using anti-inflammatory agents, preventing fibroblast formation by activating tissue plasminogen activators, and using physical barriers [4]. Anti-adhesive materials function as barriers between tissues during scar healing in tissues, and prevent adhesion band formation [5]. They are widely used to mechanically separate adjacent surfaces in clinical fields; however, they slow the healing process by disturbing cellulose deposition [6]. Furthermore, they should be degraded, absorbed, or removed after tissues are completely healed to prevent long-term physiological harm. In current use, the anti-adhesive materials are based on oxidized regenerated cellulose, sodium carboxymethylcellulose (CMC), dextran, and hyaluronate (HA), which are organic in composition, and polyethylene glycol (PEB), poloxamer, and Gore-tex, which are synthetic polymers [7].

Commercially, there are several types of anti-adhesion barriers, each with specific strengths and weaknesses. Anti-adhesion barriers formulated as solutions or gels are easy to apply and have high viscosities. However, they slide down due to gravity, and present difficulty in determining functionality as a barrier [8]. On the other hand, barriers formulated as films or membranes can cover wide surgical areas, but they are difficult to handle and often require suturing to allow tissue fixation. Another issue with these barriers is simultaneously keeping them in place as organs move and preserving their peristaltic functionality [9,10,11,12].

To compensate for these drawbacks, the Collabarrier^®^ with type 1 collagen (Dalim Center Co., Ltd., Seoul, Republic of Korea; Product No. 15-1572), a hydrophilic, biocompatible, and biodegradable material in the form of a gel, was created. Type I collagen is an extracellular matrix protein which is found in tissues such as skin, tendon, bone, and blood vessels [13]. Type I collagen gels have significantly increased autofluorescence and cross-linking, and they are resistant to enzymatic degradation, which eventually delays fibroblast invasions, thereby preventing adhesions [14]. They have been shown to have anti-adhesive properties, and to be safe in previous clinical trials. The present study examined the use of a type 1 collagen-based anti-adhesive gel in preventing postsurgical adhesion in the uterine cavity.

The primary objective was to compare the incidence rate of postoperative intrauterine adhesions with type 1 collagen gel to that of control anti-adhesives. This was based on the hypothesis that type 1 collagen gels are not inferior compared to other anti-adhesives. The secondary objective was to further compare the type and intensity of the postoperative intrauterine adhesions between the two groups to affirm the efficacy of type 1 collagen gel. Finally, as the safety assessment was also an important objective, we examined any adverse events that may occur concerning the procedure or the materials.

## 2. Materials and Methods

The study was assigned the clinical trial registration number Korea Clinical Trial Registry 0002946 (or KCTR0002946). The Clinical Research Information Service approved it on 22 June 2018.

**Figure 1:**
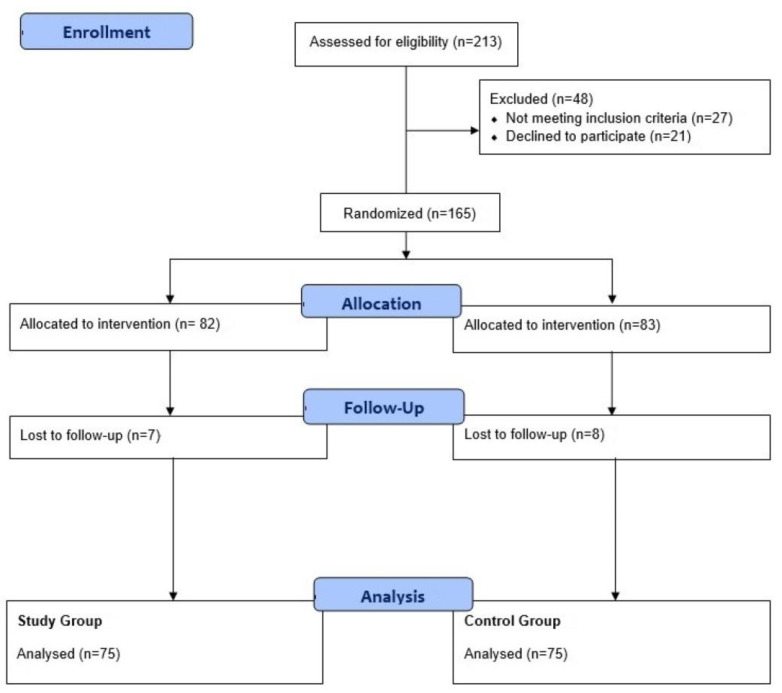
The flow diagram in enrolling patients.

Between November 2017 and February 2020, 213 patients were consecutively screened, of whom 48 patients were excluded because 27 were screened before the government’s approval and 21 patients declined to participate. A total of 165 patients were randomly assigned to two groups. Some 7 and 8 patients were lost to follow-up in the study and control groups, respectively. Therefore, 150 patients were included in the analysis (Figure 1). Written informed consent was obtained from participants before entry into the study. The study was approved by the Institutional Review Board of each institution (1-2017-0057 in Yonsei University, College of Medicine, Seoul, Republic of Korea).

The inclusion criteria included females older than 20 years old, suspected of having gynecological issues, who planned to undergo therapeutic resectoscopy, and who provided informed consent. The gynecological issues to be treated included submucosal myoma, endometrial polyps, uterine septa, endometrial hyperplasia, intrauterine adhesion, and abnormal uterine bleeding. Patients who planned to insert a hormone-secreting intrauterine device and had been treated with an agonist for gonadotropin-releasing hormone within 3 months of the study were excluded, as were those who required postoperative hormonal therapy.

### 2.1. Pre-Surgery Protocol

When a patient was screened and deemed suitable for the study, they were assigned to a treatment group after randomization. During the study, 150 women were randomized to one of two groups: the type 1 collagen gel group (Collabarrier^®^, Dalim Center Co., Ltd.) (study group; N = 75) or the HA and CMC gel group (control group; N = 75). An individual randomly assigned patients to the two treatment protocols with a 1:1 ratio. Random allocations were made based on the “PROC-PLAN” command in SAS, version 9.4 (SAS Institute, NC, USA), using the permuted block randomization method to prevent selection bias. The demographic parameters that were recorded included age, body mass index (BMI), body temperature, blood pressure, pulse rate, physical examination, pregnancy test, and laboratory examination, including red blood cell count, white blood cell count, segment neutrophil count, lymphocyte count, monocyte count, eosinophil count, basophil count, platelet count; hemoglobin, hematocrit, glucose, blood urea nitrogen, creatinine, total protein, albumin, total bilirubin, aspartate aminotransferase, alanine aminotransferase, alkaline phosphatase, gamma-glutamyl transferase (γ-GT), total cholesterol, sodium, potassium, chloride, and calcium levels; prothrombin and activated partial thromboplastin times; and urine analysis. Personal medical, smoking, and alcohol histories were also recorded.

### 2.2. Surgery Protocol

Hysteroscopies were performed on an inpatient basis at each institution by assigned gynecologists who are all experts in this surgery for more than 10 years. To minimize contamination of the endometrial cavity by vaginal bacteria, all examinations were performed after cleaning the vaginal cavity with chlorhexidine. The hysteroscopies were performed with a rigid 0° Olympus hysteroscope, with saline solution (NaCl 0.9%) as the distension medium, under anesthesia. Intrauterine cavities were inspected with hysteroscopy by surgeons, and surgeries were performed according to their decision. After resectoscopy, the antiadhesive materials were randomly allocated, and they were inserted using a thin, long, patent catheter into upper one third area of the uterine cavity.

### 2.3. Post-Surgery Follow-Up

One-week post-surgery, the patients were followed up to evaluate any adverse events due to the surgery. Adverse events (AEs) were defined as any unfavorable and unintended signs, symptoms, or diseases that were temporally associated with product use but did not necessarily have a causal relationship with the product. On the other hand, adverse device effects (ADEs) were defined as any serious adverse effects on health or safety or any life-threatening problem or death caused by or associated with the product. After applying the anti-adhesive materials, a second-look hysteroscopy was performed 4 weeks after the primary surgery to evaluate postoperative intrauterine adhesion. The intrauterine cavity was photographed during the second-look hysteroscopy. In addition, the presence and severity of intrauterine adhesions were assessed by an independent assessor.

### 2.4. Criteria and Method for Evaluating the Effectiveness

The types of adhesion were scored based on the Classification of Intrauterine Adhesions as follows: No adhesion (score, 0), Filmy (score, 1), Filmy and Dense (score, 2), Dense (score, 4).

### 2.5. Evaluation Variable for Primary Outcomes

An independent assessor evaluated the severity and scores of the intrauterine adhesions from the cavity pictures of the second-look hysteroscopy after the information for each patient was blinded. Adhesion was diagnosed when the score was ≥1. The incidence rate of intrauterine adhesion was calculated as the ratio of patients with postoperative intrauterine adhesion to the total number of enrolled patients.

### 2.6. Evaluation Variables for Secondary Outcomes

The mean scores of the intrauterine adhesion severities and the mean adhesion extent in the uterine cavity from the second-look hysteroscopy were assessed by one independent assessor. The extent of cavity involvement was scored based on the Classification of Intrauterine Adhesions as follows: 0 intensity (score, 0), less than 1/3 intensity (score, 1), between 1/3 and 2/3 intensity (score, 2), greater than 2/3 intensity (score, 4).

### 2.7. Statistical Analysis

An independent two-sample *t*-test or Wilcoxon rank sum test was used for continuous variables’ baseline characteristics. In contrast, the chi-square or Fisher’s exact test was used for categorical variables. To calculate the percentages of intrauterine adhesions after 4 weeks of resectoscopy, we presented the percentages of cases with postoperative intrauterine adhesions according to each group. Moreover, sub-groups were made according to the presence of intrauterine adhesions for further analysis, and statistical significance within each group was tested using the Cochran–Mantel–Haenszel test or Fisher’s exact test. One independent assessor evaluated the types and extent of postoperative intrauterine adhesions 4 weeks after the intrauterine surgery. The number of patients, mean values, standard deviations, median values, and minimum and maximum values were shown as descriptive statistics. Statistical significance between the groups was tested using an independent two-sample *t*-test or Wilcoxon rank sum test. Statistical analysis was performed using SPSS Statistics for Windows, version 22.0 (SPSS Inc., Chicago, IL, USA). Statistical significance was set at a *p*-value of < 0.05.

## 3. Results

Patients’ baseline characteristics are presented in Table 1. There were no significant differences in patient characteristics between groups. Four cases of others in diagnosis were two cases of uterine cervical stenosis and two cases of vaginal discharges. The primary objective was to evaluate the incidence rate of intrauterine adhesion using second-look hysteroscopy to verify the efficacy of the type 1 collagen gel (Collabarrier^®^). Women with adhesion scores >1 point were included when calculating the rate of intrauterine adhesion. Based on the results inferred by the independent assessor, the postoperative intrauterine adhesion incidence rates were 18.67% (14/75) and 13.33% (10/75) in the study and control groups, respectively (Table 2). Therefore, the difference in postoperative intrauterine adhesion rates between the two treatment groups was 1.78%, with an upper limit of 97.5% and a one-sided confidence interval of 14.54%. As this value was lesser than the non-inferiority threshold value of 15%, we could verify that the study group was not inferior to the control group.

Subsequent analysis was performed by stratifying each group according to the presence or absence of preoperative intrauterine adhesions, and further, by the formation of postoperative intrauterine adhesion in each group. Among the study group patients, 66.67% (2/3) had preoperative intrauterine adhesions, whereas 16.67% (12/72) only developed the adhesions postoperatively. In the control group, postoperative intrauterine adhesions were seen in 0.00% (0/4) of patients preoperatively, and these developed only post-surgery in 14.08% (10/71) of patients. Therefore, the subgroup analysis based on the preoperative presence of intrauterine adhesion did not show any statistical significance (Table 2). There were no significant differences in the postoperative intrauterine adhesion incidence rate of the two groups, proving that the type 1 collagen-based anti-adhesion gel is not inferior compared with the control.

We evaluated the secondary objective by comparing the type of adhesion formed in the uterine cavity a month after the intrauterine surgery. The results of the analysis of the postoperative intrauterine adhesions performed by an independent assessor were classified as scored in the methods. No adhesions were seen in 81.33% (61/75) of patients in the study group versus 86.67% (65/75) of patients in the control group. Filmy adhesions were observed in 14.67% (11/75) of patients in the study group versus 13.33% (10/75) of patients in the control group. Filmy and dense adhesions were seen in 4.00% (3/75) of patients in the study group compared with 0.00% (0/75) of patients in the control group. Finally, dense adhesions were not observed in either group. The mean score of the adhesion types in the study group was 1.19 compared with 1.14 in the control group. Therefore, there were no statistical inter-group differences in the percentages of adhesion types and the mean scores for both groups (Table 3).

An independent assessor also evaluated the extent of the cavity involved (or intensity) of intrauterine adhesions a month after the initial surgery, as described in the methods. The percentages of each group based on the intensity of the adhesions were as follows: 0 intensity adhesions were seen in 81.33% (61/75) of patients in the study group versus 86.67% (65/75) of patients in the control group. Adhesions with <1/3 intensity were seen in 13.33% (10/75) of patients in the study group and 13.33% (10/75) of patients in the control group. Adhesions between 1/3 and 2/3 intensity were observed in 5.33% (4/75 patients) of the study group and 0.00% (0/75 patients) of the control group. Finally, adhesions with an intensity >2/3 were not observed in either group.

Furthermore, the mean score of the extent of the intrauterine adhesions was 1.20 in the study group and 1.14 in the control group. Finally, no statistical inter-group differences were observed in the adhesion intensity scores. (Table 3).

Overall, 102 patients (58.29%) had a medical history that included details of prior illnesses and surgeries. These were evaluated to observe the outcomes according to the presence of a previous medical history, but the difference found was not statistically significant. Medical histories were classified based on the System Organ Classes (SOC) of the Medical Dictionary for Regulatory Activities (MedDRA); the most frequently observed category was “reproductive system” (40 patients with 45 incidences) and “breast disorders” (20 patients with 22 incidences). The personal history, which includes information on allergies and ailments, was recorded in 84 patients with 136 incidences. The two groups did not show any statistically significant different prevalence. When the present personal history was classified based on the SOC of MedDRA, the most frequently ranked category was “reproductive system and breast disorders,” noted in 33 patients with 38 incidences. However, it did not have a significant impact on the outcome. There were no significant changes from visit 1 to visit 4 in the laboratory, evaluations except in one case, where the γ-GT level at visit 4 was clinically significant.

No statistically significant differences were noted between the two groups in the AEs and serious AEs (Table 4). Moreover, as the patients suffering from serious AEs were marked to recover, both surgical options had a good prognosis overall.

Overall, 39 patients were reported to have 53 AEs, of which 22 (29.33%) and 17 (22.67%) patients from the study and control groups had 32 and 21 AEs, respectively. Seven patients experienced seven serious AEs (Table 5). Patients with serious AEs were categorized as “patients who need to be admitted or who need an admission period extension” in two cases (two cases in the study group), and “patients managed to address another medical problem” in five cases (three cases in the study group, two cases in the control group). Serious AE status was reported as “recovered” in one case (one case in the study group, zero cases in the control group), “in the process of recovery” in five cases (three cases in the study group, two cases in the control group), and “unknown recovery status” in one case (one case in the study group, zero cases in the control group); none of them were reported as “did not recover” or “dead.”

ADEs were recorded in four events in four patients (2.67%), of which three patients (4.00%) were from the study group, and one patient (1.33%) was from the control group. All ADEs were judged as “mild,” which did not require intervention, and the patients recovered completely. The study group included one case of procedural hemorrhage, one of procedural pain, and one of vaginal discharge. In contrast, the control group showed one case of vaginal hemorrhage that spontaneously regressed. There were no serious ADEs (Table 5). Without any medical intervention, three cases (two cases in the study group, one case in the control group) “recovered,” and one patient (one case in the study group) was “in the process of recovery”. No statistically significant differences in ADEs and serious ADEs were noted between the two groups (Table 4).

In addition to the analysis of the ADEs, laboratory tests were performed to monitor safety. Analyzing changes in the laboratory parameters before and after applying the materials into uterine cavity did not reveal any differences between the groups. The healthy levels or non-clinically significant parameters before the procedure did not become clinically significant.

Therefore, in evaluating the safety of type 1 collagen gel, there were no statistical differences between the study and control groups. Notably, a few cases of AEs were reported as “few associations”; however, most AEs were reported as irrelevant to the medical device. All adverse device events were categorized into mild cases with no requirement of a specific intervention, and they were reported to be “recovered” or “in the process of recovery.” There were no serious medical device-related AEs. Therefore, type 1 collagen gel proved safe as an anti-adhesive after intrauterine surgery.

## 4. Discussion

The incidence rate of postoperative intrauterine adhesions did not show any significance between the Collabarrier^®^ and the control groups. The frequency and mean scores of the type and intensity of adhesions and AEs, serious AEs, adverse device effects, and serious adverse device effects were not significantly different between the two groups. These results demonstrate the beneficial effects of Collabarrier^®^ in terms of effectiveness and safety.

Since postoperative adhesions induce various complications such as infertility, amenorrhea, and recurrent pregnancy loss; several trials have been conducted to minimize postoperative adhesions through minimal tissue manipulation during surgery through the use of anti-inflammatory agents, the activation of tissue plasminogen activators to prevent fibroblast formation, and the use of physical barriers [4]. The anti-adhesive materials that function as tissue barriers during tissue healing should be degraded, absorbed, or removed when this process is completed. Materials should be degraded and induce no physiological harm. Recently created anti-adhesive materials such as CMC, dextran, HA, polyethylene glycol (PEB), poloxamer, and Gore-tex are offered in solutions and gels [7]. These are easy to apply and have thick viscosities. However, they run away from the wound due to gravity, and absorption and excretion occur rapidly; thus, their duration as a physical barrier is hard to determine [8]. In contrast, films and membranes are applicable in the broad surgical field. However, in some cases, the materials require fixation to the tissues via suturing, and may not always be accurately placed due to organ peristalsis [9,10,11,12].

To compensate for these drawbacks, Collabarrier^®^ was made with type 1 collagen in gels with hydrophilic, biocompatible, and biodegradable characteristics, to act as a deep wound-covering material. It has been shown to have anti-adhesive properties and to be safe in previous clinical trials. It has already been approved as a surgical wound-covering material in thyroid surgery to prevent adhesions [15]. Moreover, it is cheaper than other anti-adhesive materials, so it can reduce hospital costs.

This study’s primary objective was to examine the postoperative adhesion rate between the two groups. The lack of difference in this rate showed that the type 1 collagen gel (Collabarrier^®^) was not inferior to the HA and CMC gels in preventing postoperative intrauterine adhesions. Moreover, the secondary assessment of the type and intensity of the postoperative intrauterine adhesion showed no statistically significant differences between the two groups, thereby demonstrating the type 1 collagen gel to be an effective anti-adhesive. In evaluating the safety of type 1 collagen gel, there were no statistical differences in the adverse device events between the two groups. Moreover, all the events were in various stages of recovery by the end of the trial. Therefore, in evaluating the safety of type 1 collagen gel, there were no statistical differences between the study and control groups.

A few cases of AEs were reported as “few associations”; however, most AEs were reported as irrelevant to the medical materials used. All adverse events were categorized into mild cases with no requirement of a specific intervention, and they were reported to be “recovered” or “in the process of recovery”. There were no serious medical device-related AEs.

This study had some limitations. First, this study was multi-centered; however, it was exclusively focused on Korean women. Secondly, during the postoperative assessment, we asked the operators conducting the second-look hysteroscopies to take pictures of the intrauterine cavity, especially focusing on postoperative adhesions if there were any. Therefore, the independent assessors who evaluated the postoperative intrauterine adhesions had to make judgments with only these few pictures from the second-look hysteroscopies. Therefore, we could not say that full inspections of the intrauterine cavity were properly carried out. Third, we should have not performed the second look hysteroscopy on five patients who were pathologically diagnosed with uterine malignancy. The use of hysteroscopy in already confirmed endometrial cancer is still controversial. Some studies have reported that spillages of washing fluids into the pelvic cavity may impact negatively on the prognosis of the disease, whereas others report that hysteroscopy does not result in cancer cell spreading into the peritoneal cavity [16].

Through this trial, we demonstrate that type 1 collagen gel can be used effectively and safely in intrauterine surgery to minimize postoperative adhesions, eventually decreasing the prevalence of infertility, secondary amenorrhea, and recurrent pregnancy loss in reproductive women.

## Figures and Tables

**Table 1:** Baseline characteristics of the participants.

	Study Group (N = 75)	Control Group (N = 75)	*p*-Value
Age (years)	41.22 (7.312)	42.47 (6.998)	0.284 ^a^
BMI (kg/m^2^)	22.48 (6.263)	23.21 (7.265)	0.423 ^a^
Smoking within a year			0.681 ^b^
Yes	4 (5.33)	2 (2.67)	
No	71 (94.67)	73 (97.33)	
Drinking within a year			0.866 ^c^
Yes	26 (34.67)	27 (36.0)	
No	49 (65.33)	48 (64.0)	
Laboratory test			0.245 ^b^
Hemoglobin ≤ 10.0 g/dL	0 (0.0)	3 (4.0)	
Hematocrit ≤ 30.0%	0 (0.0)	3 (4.0)	
Diagnosis			0.373 ^b^
Submucosal myoma	22 (22.7)	20 (20.6)	
Endometrial polyp	59 (60.8)	63 (64.9)	
Uterine septum	1 (1.0)	0 (0.0)	
Endometrial hyperplasia	5 (5.2)	6 (6.2)	
Intrauterine adhesion	2 (2.1)	1 (1.0)	
Abnormal uterine bleeding	4 (4.1)	7 (7.2)	
Others	4 (4.1)	0 (0.0)	

N (%): Number of participants (percentages); BMI: body mass index; Diagnosis: all diagnoses (≥1 diagnosis for one participant) made were included; Laboratory test: Serum blood tests performed on visit 1; ^a^ Independent *t*-test, ^b^ Fisher’s exact test, ^c^ Pearson’s chi-square test.

**Table 2:** Presence of intrauterine adhesion before and after therapeutic resectoscopy.

Intrauterine Adhesion		Study Group (N = 75)	Control Group (N = 75)	*p*-Value
Pre-op	Post-op			
Yes	Yes No	2 (66.67) 1 (33.33)	0 (0.0) 4 (100.0)	
No	Yes No	12 (16.67) 60 (83.33)	10 (14.08) 61 (85.92)	
Presence of intrauterine adhesion	Yes	14	10	0.946

Cochran–Mantel–Haenszel test.

**Table 3:** Presence and characteristics of intrauterine adhesions after second-look hysteroscopy.

	Study Group (N = 75)	Control Group (N = 75)	*p*-Value
Type of adhesions (score)			0.279 ^a^
No adhesion (0)	61 (81.3)	65 (86.7)	
Filmy (1)	11 (14.7)	10 (13.3	
Filmy and Dense (2)	3 (4.0)	0 (0.00)	
Dense (4)	0 (0.0)	0 (0.00)	
Type of adhesions (value)			0.6791 ^b^
Mean (SD)	1.19 (0.47)	1.14 (0.35)	
Median	1.00	1.00	
Min, Max	1.00, 3.00	1.00, 2.00	
The extent of cavity involvement (score)			0.176 ^a^
0 (0)	61 (81.3)	65 (86.7)	
<1/3 (1)	10 (13.3)	10 (13.3)	
1/3–2/3 (2)	4 (5.3)	0 (0.00)	
>2/3 (4)	0 (0.00)	0 (0.00)	
The extent of cavity involvement (value)			0.6581 ^b^
Mean (SD)	1.20 (0.50)	1.14 (0.35)	
Median	1.00	1.00	
Min, Max	1.00, 3.00	1.00, 2.00	

The types of adhesion were scored as 0 = No adhesion, 1 = Filmy, 2 = Filmy and Dense, 4 = Dense to evaluate the severity of intrauterine adhesion; the extent of cavity involvement was scored as 0 = 0, 1 = <1/3, 2 = 1/3–2/3, 4 = >2/3, based on the Classification of Intrauterine Adhesions scoring system. ^a^ Fisher’s exact test, ^b^ Wilcoxon rank sum test.

**Table 4:** Prevalence of adverse events and adverse device effects in the two groups.

	Study Group (N = 75)		Control Group (N = 75)		*p*-Value
	N (%)	E	N (%)	E	
Adverse events	22 (22.68)	32	17 (18.28)	21	0.4528 ^a^
Serious adverse events	5 (5.15)	5	2 (2.15)	2	0.4451 ^b^
Adverse device effects	3 (4.00)	3	1 (1.30)	1	0.620 ^b^
Serious adverse device effects	0 (0.00)	0	0 (0.00)	0	-

^a^ chi-square test; ^b^ Fisher’s exact test; - not analyzed.

**Table 5:** Serious adverse events and adverse device effects according to systemic organ classes.

	Study Group (N = 75)		Control Group (N = 75)		Total (N = 150)	
	N (%)	E	N (%)	E	N (%)	E
**Serious adverse events**	5 (6.67)	5	2 (2.67)	2	7 (4.67)	7
Neoplasms benign, malignant, and unspecified	3 (4.00)	3	2 (2.67)	2	5 (3.33)	5
Endometrial cancer	2 (2.67)	2	1 (1.33)	1	3 (2.00)	3
Endometrial adenocarcinoma	1 (1.33)	1	0 (0.00)	0	1 (0.67)	1
Uterine cancer	0 (0.00)	0	1 (1.33)	1	1 (0.67)	1
Respiratory, thoracic, and mediastinal disorders	1 (1.33)	1	0 (0.00)	0	1 (0.67)	1
Pulmonary edema	1 (1.33)	1	0 (0.00)	0	1 (0.67)	1
Nervous system disorders	1 (1.33)	1	0 (0.00)	0	1 (0.67)	1
Mononeuropathy	1 (1.33)	1	0 (0.00)	0	1 (0.67)	1
**Adverse device effects**	3 (4.00)	3	1 (1.33)	1	4 (2.67)	4
Injury, poisoning, and procedural complications	2 (2.67)	2	0 (0.00)	0	2 (1.33)	2
Procedural hemorrhage	1 (1.33)	1	0 (0.00)	0	1 (0.67)	1
Procedural pain	1 (1.33)	1	0 (0.00)	0	1 (0.67)	1
Reproductive system and breast disorders	1 (1.33)	1	1 (1.33)	1	2 (1.33)	2
Vaginal discharges	1 (1.33)	1	0 (0.00)	0	1 (0.67)	1
Vaginal hemorrhage	0 (0.00)	0	1 (1.33)	1	1 (0.67)	1

N (%): Number of patients (percentages); percentages were calculated according to each group; E: Number of events. Coding was performed based on MedDRA SOC (system organ class) and PT (preferred term).

## Data Availability

The de-identified data supporting this study’s findings are available on request to bona fide researchers who provide a methodologically sound proposal. The data will be made available 24 months after the study’s completion. Proposals should be directed to the corresponding author. Data requesters will need to sign a data access agreement to gain access.
